# A comparative study on person-centered care practice between public and private General Hospitals in Addis Ababa, Ethiopia

**DOI:** 10.3389/frhs.2024.1482363

**Published:** 2024-12-18

**Authors:** Mierage Ali, Tesfaye Getachew Charkos

**Affiliations:** ^1^ Department of Public Health, Yekatit 12 Hospital Medical College, Addis Ababa, Ethiopia; ^2^ School of Public Health, Adama Hospital Medical College, Adama, Ethiopia

**Keywords:** healthcare, patient-physician interaction, magnitude, associated factors, Ethiopia

## Abstract

**Background:**

Person-centered care practice has not yet been fully adopted in low- and middle-income nations such as Ethiopia. It focuses on improving several areas of patient-physician interaction. Despite Ethiopia's rapid growth in healthcare facilities, there is insufficient data available on care practices.

**Method:**

A facility-based comparative cross-sectional study was conducted in selected public and private general hospitals in Addis Ababa from May 26 to July 26, 2023. A multistage sampling technique was employed to select the study participants. The data were collected using an interviewer-administered structured questionnaire, entered into Epi Info-7, and exported to SPSS version 27 for analysis. Bivariate and multivariate logistic regression analyses were performed to identify significant factors associated with care practices.

**Results:**

A total of 848 patients were involved, with a response rate of 99.5%. The overall magnitude of good care practice was 52.8%, with 34.8% in public hospitals and 70.9% in private hospitals. Factors associated with good care practices in private hospitals included hospital attractiveness (AOR: 3.2; 95% CI: 1.6–6.5), ease of access to services (AOR: 12.1; 95% CI: 6.2–23.3), and privacy of access and care (AOR: 10.89; 95% CI: 5.60–21.19). In contrast, factors associated with good healthcare practices in public hospitals were perceived intimacy with the provider (AOR: 8.85; 95% CI: 4.50–17.43), privacy in accessing care (AOR: 12.1; 95% CI: 6.62–22.16), and the provision of medication information (AOR: 4.39; 95% CI: 2.40–8.03).

**Conclusion:**

Overall, 52.8% of participants rated person-centered care practices as good, with private hospitals in Addis Ababa (70.9%) demonstrating a higher prevalence of person-centered care practices compared to public hospitals (34.8%). The factors associated with healthcare practices in both public and private hospitals include hospital type, hospital attractiveness, ease of access to services, privacy in accessing care, perceived intimacy with the provider, and the provision of medication information. We recommend targeted improvements in public hospitals to enhance the quality of PCC.

## Introduction

The Institute of Medicine (IOM) defines person-centered care (PCC) as care that respects and responds to individual patient preferences, needs, and values, ensuring that these values guide all clinical decisions, and it is identified as one of six essential goals for healthcare improvement (1). Person-centered care is holistic and empowering, recognizing and prioritizing the unique needs and preferences of each patient (2). The Picker Institute further explained that PCC has eight dimensions: (1) respect, (2) coordination and integration of care, (3) information, communication, and education, (4) physical comfort, (5) emotional support, (6) family and friends' involvement, (7) transition and continuity, and (8) access to care (3). Carl Rogers introduced person-centered care in the 1940s, leading to the development of advanced models and their application in various fields of practice (4). The World Health Organization (WHO) has emphasized the importance of the "PCC" in providing quality care for patients with chronic diseases, with patients and healthcare professionals as key components (4).

Despite its advantages, care encounters significant global implementation challenges, with inconsistent integration into clinical practice, even in developed countries (2). Studies indicate that barriers to implementing person-centered care include time constraints, heavy workloads, resistance to change, lack of organizational support, limited involvement of front-line staff, and inadequate resources (5). Similarly, evidence suggested that the barriers to care practice include time constraints, patient characteristics, providers' reluctance to relinquish control, and poor communication skills (6). A study found that poor PCC implementation in sub-Saharan Africa is due to provider issues, health system structure, and the broader socioeconomic environment (2).

Ethiopian healthcare tends to be more biological than biopsychosocial, with 71% of healthcare professionals lacking compassion and respect, leading to 30% of patients expressing dissatisfaction with the services (3). A study in Addis Ababa revealed that 49% of patients viewed the care as person-centered, with private hospitals showing a higher rate of 70.2% (7). This study aimed to assess and compare healthcare practices among public and private general hospitals in Addis Ababa, addressing research gaps in Ethiopia.

## Methods

### Study area and period

The study was conducted in general hospitals in Addis Ababa, Ethiopia, from May 26 to July 26, 2023. The city has a total of 52 hospitals, including three nongovernmental organizations (NGOs), three owned by the defense forces and the police, and 35 private hospitals. According to the Central Statistical Agency of Ethiopia (CSA) population forecast for 2022, Addis Ababa has a total population of 3,859,999, with 1,882,000 males.

### Study design

A facility-based cross-sectional study design was conducted.

### Source population

All admitted inpatients in public and private general hospitals in Addis Ababa during the study period were included in the study.

### Study population

Randomly selected admitted inpatients from the chosen general hospitals between May 26 and July 26 were included in the study.

### Inclusion criteria

Patients over 18 years of age, admitted to selected public and private hospital wards for 24 h or more, and mentally capable of providing informed responses, were included in the study.

### Exclusion criteria

In this study, unconscious patients, those with impaired cognitive or communication abilities, and ICU patients were excluded from the analysis.

### Sample size and sampling procedure

The sample size was determined using the double population proportion formula, with the following statistical assumptions: a 95% confidence interval (CI) and a proportion of person-centered care (PCC) practice in public hospitals (P1 = 60.9%) based on a study conducted in Wollo (8), and the proportion of PCC practice at private hospitals (P2 = 70.2%) from the study conducted in Addis Ababa (7) [Alpha (*α*) type 1 error; *β* is a type 2 error; power = 80%; confidence level = 1.96. Where *Zα*/2 is the critical value of the normal distribution at *α*/2 (for a 95% confidence level, for example, *α* is 0.05 and the critical value is 1.96) and *Zβ* is the critical value of the normal distribution at *β* (for example, *β* is 0.2 for 80% power) and the critical value is 0.84), and p1 and p2 are the expected sampling proportions of the two groups.

$$n=\frac{{\left(Z_{\frac{\alpha }{2}}+Z_{\beta }\right)}^{2}*(\;p_{1}(1-p_{1})+p_{2}(1-p_{2}))}{{\left(\;p_{1}-p_{2}\right)}^{2}}=406$$

**Figure 1:**
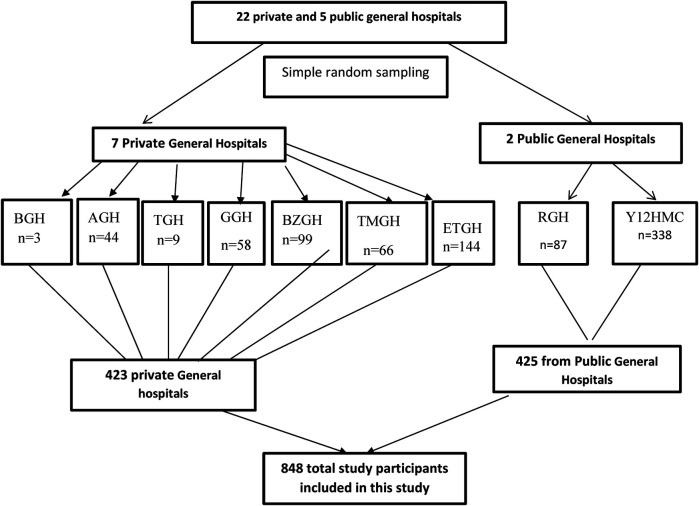
Schematic representation of the sampling selection procedure.

After accounting for a 5% nonresponse rate, the final sample size was set at 852, with 426 patients from private hospitals and 426 from public hospitals. Out of 27 general hospitals in the city (5 public and 22 private), two public hospitals (Ras-Desta Hospital and Yekatit 12 Hospital Medical College) and seven private hospitals (Betel General Hospital (BGH), Amen General Hospital (AGH), Tezena General Hospital (TGH), Grum General Hospital (GGH), Betezata General Hospital (BZGH), Teklehymtnot General Hospital (TMGH), and Ethio-Tebib General Hospital (ETGH)) were selected (30% of each group) using a lottery method. The sample size was proportionally allocated based on the monthly average of hospitalized patients at each hospital, and systematic random sampling was employed to identify study participants (Figure 1).

### Study variables and measurements

In this study, the dependent variable was person-centered healthcare practices. The independent variables included sociodemographic factors (age, gender, educational status, marital status, income, and insurance status), organizational characteristics (hospital type, welcoming environment, ease of service access, noise levels, aesthetic appeal, privacy, and information on care plans, safety alerts, and diet), and healthcare provider factors (perceived intimacy, physician competency, and self-assessed clinical knowledge).

### Data collection method and tool

#### Person-centered climate questionnaire-patient

The Patient-Centered climate questionnaire (PCQ-P), originally developed in Swedish, assesses patients' perspectives on the care environment within healthcare facilities. It comprises 17 items across three dimensions of person-centered care: safety, everydayness, and hospitality. The Swedish study reported satisfactory Cronbach's alpha values for the total scale (0.93) and its subscales safety (0.94), everydayness (0.80), and hospitality (0.64). Overall, the person-centered climate questionnaire is a valid and reliable tool for evaluating the extent of person-centeredness in hospital environments (8). The English version of the PCQ-P was also validated in an Australian study (9).

Since no Amharic version was available, the English version was translated into Amharic using the forward-backward translation method. The Amharic version demonstrated high reliability, with Cronbach's alpha values of 0.96 for the total scale and 0.88, 0.89, and 0.91 for the subscales of safety, everydayness, and hospitality, respectively. A pretest was conducted on 5% of the sample at St. Peter's Specialized Hospital to ensure the translation's accuracy and reliability.

### Operational definition

A climate of safety is indicated by accessible and competent staff who respond quickly and a clean and well-organized physical environment (10).

Everyday climate refers to experiences of a deinstitutionalized environment that contains aspects of familiarity and everydayness and being home-like (10).

Hospitality refers to the reception and friendliness of local people, who both make you feel welcome and receive the best treatment and care (8).

Welcoming space to patient and family, Sound and noise, Ease to access services within the institution, Beauty and external appearance, Privacy to access care, Communication on the plan of care, Medication, Diet and Safety, Perceived intimacy with the provider, for the above factors Patients were classified as having a “good” perception if they responded very good and good and a “poor” perception if they responded either neutrally, poorly or very poorly (11).

Person-centered care practices: were measured with a 17-item structured questionnaire with responses structured on a five-point scale. Respondents were categorized as experiencing “good” person-centered care practices (PCC) if they scored above or equal to the mean score of 3.46, while those who scored below the mean were categorized as experiencing “poor” PCC.

### Data analysis

Descriptive statistics such as frequencies and percentages were used for qualitative variables. The internal consistency of the PCQ-P was calculated using Cronbach's *α* coefficient. Bivariate and multiple binary logistic regression analyses were used to determine the associations between independent and dependent variables. The crude odds ratio (COR) and adjusted odds ratio (AOR) were calculated. To determine the factors significantly associated with the PCC, the odds ratio (OR) at the 95% CI was determined using multivariable logistic regression analysis. The Hosmer and Lemeshow tests were used to test the goodness of fit. Multicollinearity was determined using a VIF cutoff point >10. An adjusted odds ratio with a *p*-value < 0.05 was used to report the significant factors associated with PCC. All analyses were performed using the Statistical Package for Social Science (SPSS version 25).

## Results

### Sociodemographic characteristics of the participants

This study included 848 participants from public and private hospitals, with a response rate of 99.5%. Most participants were female (58%) in private hospitals and male (54.1%) in public hospitals. The average age was 44.6 ± 14.3 years for private hospital patients and 47 ± 13.8 years for public hospital patients. Regarding marital status, 52% of participants in private hospitals and 70.8% in public hospitals were married. Monthly incomes ranged from none to 27,000 ETB (Table 1).

**Table 1:** Sociodemographic characteristics of the respondents at the public and private general hospitals of Addis Ababa, Ethiopia, 2023 (*N* = 848).

Variables	Category	Private hospitals (*n* = 423)		Public hospitals (*n* = 425)	
		Frequency	Percentage (%)	Frequency	Percentage (%)
Gender	Female	251	59.3	195	45.9
	Male	172	40.7	230	54.1
Age	18–35	165	39.0	105	24.7
	36–45	125	29.6	108	25.4
	45+	133	31.4	212	49.9
Marital status	Single	97	22.9	65	15.3
	Married	220	52.0	301	70.8
	Others	106	25.1	59	13.9
Educational level	No education	40	9.5	40	9.4
	Primary (1–8)	126	29.8	153	36.0
	Secondary (9–12)	186	44.0	149	35.1
	Above secondary	71	16.8	83	19.5
Insurance status	Insured	17	4.0	266	62.6
	Noninsured	406	96.0	159	37.4
Income	<5,000	160	37.8	181	42.6
	5,000–10,000	200	47.3	237	55.8
	10,000+	63	14.9	7	1.6

### Organizational related factors

A majority of participants from private hospitals (71.4%) and public hospitals (63.8%) perceived the hospital environment as friendly. Accessibility of services was reported as easy by only 34.8% of participants from public hospitals, compared to 76.1% from private hospitals. Most clients from both public (79.7%) and private hospitals (71.3%) reported that there was no disruptive noise on the hospital premises.

Most participants from private hospitals, 302 (71.4%) felt that the hospital had a welcoming environment, compared to 271 (63.8%) of those from public hospitals. Additionally, 322 (76.1%) participants from private hospitals reported easy access to services, while only 34.8% of public hospital participants felt the same.

Of the respondents, 295 (69.4%) from public hospitals and 332 (78.3%) from private hospitals felt the facilities were well-maintained. Privacy was considered adequate by 74.7% of private hospital patients and 46.4% of public hospital patients. Most participants shared their views on care quality, with 352 (83.2%) from private hospitals and 332 (78.3%) from public hospitals providing feedback. Additionally, 331 private hospital patients (78.3%) and 259 public hospital patients (60.9%) felt they received sufficient information about safety alerts (Table 2).

**Table 2:** Perceived organizational factors affecting person-centered care practices at public and private general hospitals in Addis Ababa, Ethiopia, in 2023 (*N* = 848).

Variables	Private hospitals		Public hospitals	
	Poor	Good	Poor	Good
The hospital has a welcoming space and approach for patients and families	121 (28.6%)	302 (71.4%)	154 (36.2%)	271 (63.8%)
Access services easily within the institution	101 (23.9%)	322 (76.1%)	277 (65.2%)	148 (34.8%)
Disturbing sound in the compound	86 (20.3%)	337 (79.7%)	122 (28.7%)	303 (78.1%)
The hospital's external attractive status	91 (21.5%)	332 (78.5%)	130 (30.6%)	295 (69.4%)
Was there privacy to access your care	107 (25.3%)	316 (74.7%)	228 (53.6%)	197 (46.4%)
The plan of care communicated to wish you enough	71 (16.8%)	352 (83.2%)	93 (21.9%)	332 (78.1%)
Was the communication of the safety alert provided clearly	92 (21.7%)	331 (78.3%)	166 (39.1%)	259 (60.9%)
Was the information on diet communicated	112 (26.5%)	311 (73.5%)	156 (36.7%)	269 (63.3%)
Information told on medication enough	89 (21.0%)	334 (79.0%)	166(39.1%)	259(60.9%)

### Self- and physician-related factors

Approximately 61.4% of public hospital clients and 80% of private hospital clients were aware of their healthcare providers. Knowledge of their condition was reported by 284 (66.8%) public hospital participants and 348 (82.3%) private hospital participants. Three-foruth (75.4%) of private hospital participants and 71.1% of public hospital participants were aware of the available treatment options. Most participants felt well-informed about their illness, with 406 (96%) in private hospitals and 393 (92.5%) in public hospitals showing strong knowledge of their condition (Table 3).

**Table 3:** Perceived self- and provider-related factors that might affect person-centered care practices at public and private general hospitals in Addis Ababa, Ethiopia, in 2023 (*N* = 848).

Variables	Private hospitals		Public hospitals	
	No	Yes	No	Yes
Do you know each other with the physician who gave you the care	88 (20.8%)	335 (79.2%)	164 (38.6%)	261 (61.4%)
Do you think you have an awareness of your disease or case	75 (17.7%)	348 (82.3%)	141 (33.2%)	284 (66.8%)
Do you think you have an awareness of your treatment options for your disease	104 (24.6%)	319 (75.4%)	123 (28.9%)	302 (71.1%)
Do you think the physician had enough knowledge about your disease	17 (4.0%)	406 (96.0%)	32 (7.5%)	393(92.5%)

### Person-centered care at public and private hospitals

**Figure 2:**
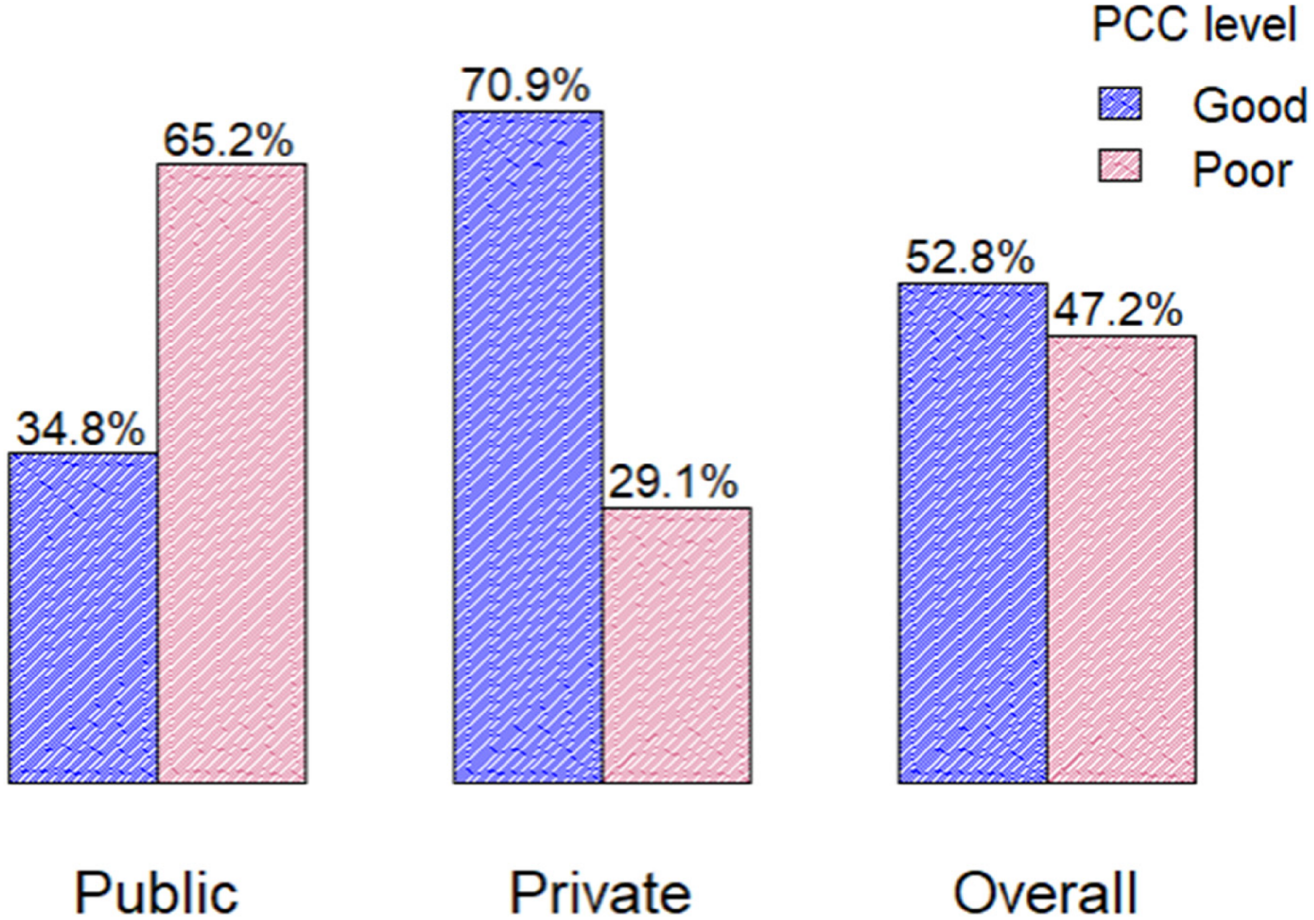
The overall PCC in public, private, and general hospitals in Addis Ababa city, Ethiopia, in 2023 (*n* = 848).

The magnitude of good person-centered care (PCC) in public general hospitals in Addis Ababa was 34.8% (95% CI: 30.3%–39.3%), compared to 70.9% (95% CI: 66.6%–75.2%) in private hospitals. Overall, 52.8% (95% CI: 49.6%–56.1%) of respondents rated the PCC practice as good (Figure 2).

### Factors associated with PCC in private hospitals

Multivariable logistic regression analysis revealed that hospital attractiveness, ease of access to services, and privacy during care were significantly associated with person-centered care (PCC) practices in private hospitals. Patients who perceived the hospital as unattractive were three times more likely to rate its PCC as poor (AOR: 3.4; 95% CI: 1.70–6.77). Those who found accessing services difficult were 13.12 times more likely to consider PCC poor (AOR: 13.12; 95% CI: 6.70–25.72) compared to those who found access easy. Additionally, patients who perceived poor privacy during care were approximately eleven times more likely to rate PCC as poor (AOR: 11.01; 95% CI: 5.70–21.27) than their counterparts (Table 4).

**Table 4:** Factors associated with person-centered care practices in private and public general hospitals (*n* = 848).

Variables	Category	Private hospital			Public hospital		
		Poor	Good	AOR (95%)	Poor	Good	AOR (95%)
Age	18–35	47	118	0.66 (0.25,1.77)	62	43	2.18 (0.98,4.86)
	36–45	46	79	0.64 (0.27,1.50)	69	39	2.01 (0.99,4.12)
	45+	30	103	1	146	66	1
Marital status	Single	35	62	2.02 (0.64,6.34)	36	29	1.01 (0.30,3.31)
	Married	63	157	1.08 (0.50,2.33)	203	98	0.61 (0.24,1.60)
	Others	25	81	1	38	21	1
Educational level	No education	9	31	0.54 (0.12,2.51)	22	18	2.35 (0.71,7.76)
	Primary	29	97	1.58 (0.59,4.25)	107	46	1.80 (0.75,4.29)
	Secondary	60	126	0.79 (0.33,1.83)	97	52	1.97 (0.86,4.56)
	Higher	25	46	1	51	32	1
Insurance status	Insured	4	13	0.22 (0.040,1.18)	173	93	0.79 (0.44,1.39)
	noninsured	119	287	1	104	55	1
Do you know each other with the physician who gave you the care	No	44	44	2.09 (0.99,4.40)	148	16	**8.77 (4.47,17.22)**
	Yes	79	256	1	129	132	1
Awareness of your disease or case	No	27	48	0.82 (0.32,2.11)	108	33	0.79 (0.36,1.78)
	Yes	96	252	1	169	115	1
Awareness of the treatment options	No	37	67	**2.63 (1.16,5.97)**	94	29	1.69 (0.760,3.77)
	Yes	86	233	1	183	119	1
Access services easily within the institution	Poor	73	28	**13.12 (6.70,25.72)**	178	75	1.43 (0.812,2.50)
	Good	50	272	1	99	73	1
Disturbing sound in the compound	Poor	30	56	1.64 (0.79,3.40)	86	36	1.08 (0.55,2.13)
	Good	93	244	1	191	112	1
The hospital's external attractive status	Poor	35	56	**3.41 (1.70,6.87)**	97	33	1.32 (0.70,2.47)
	Good	88	244	1	180	115	1
Privacy to access your care	Poor	73	34	**11.01 (5.70,21.27)**	204	24	**12.08 (6.61,22.09)**
	Good	50	266	1	73	124	1
The care communication is enough	Poor	25	46	0.80 (0.37,1.72)	72	21	1.21 (0.578,2.56)
	Good	98	254	1	205	127	1
Information told on medication enough	Poor	35	54	1.54 (0.75,3.15)	136	30	**4.41 (2.41,8.05)**
	Good	88	246	1	141	118	1

COR, crude odds ratio, AOR, adjusted odds ratio.

Bold: indicates statistical significance (*p* < 0.05).

### Factors associated with PCC in public hospitals

Similarly, in the multivariable binary logistic regression analysis for public hospitals, perceived intimacy with the provider, privacy during care, and information about medication were significantly associated with person-centered care (PCC) practices. Patients who did not know their healthcare provider were eight times more likely to rate the care as poor (AOR: 8.77; 95% CI: 4.47–17.22) compared to their counterparts. Those who perceived poor privacy in the hospital were twelve-fold more likely to rate PCC as poor (AOR: 12.08; 95% CI: 6.61–22.09) than those who perceived good privacy. Similarly, patients who felt they received inadequate information about their medication were 4.4 times more likely to rate the care as poor (AOR: 4.41; 95% CI: 2.41–8.05) compared to those who felt adequately informed (Table 4).

### Factors associated with PCC in the overall hospital

In the overall adjusted model, hospital type, perceived intimacy with the provider, ease of accessing services, hospital attractiveness, privacy during care, and medication information were significantly associated with person-centered care practices. Patients from private hospitals were 56% more likely to perceive the care they received as good for person-centered care (PCC) than those from public hospitals (AOR: 0.44; 95% CI: 0.26–0.75). Patients who did not know their healthcare provider were 4.8 times more likely to rate the PCC interaction as poor (AOR: 4.80; 95% CI: 3.07–7.50) compared to those who knew their provider. Patients who found it difficult to access services were 3.4 times more likely to rate the care as poor for PCC than those who found it easy to access services (AOR: 3.36; 95% CI: 2.24–5.03). Patients who perceived the hospital as unattractive were 1.65 times more likely to rate the care as poor for PCC (AOR: 1.65; 95% CI: 1.05–2.59) compared to those who found the hospital appealing. Patients who believed the hospital had poor privacy were 11.21 times more likely to perceive the care as poor for PCC (AOR: 11.21; 95% CI: 7.38–17.04). Patients who felt they did not receive adequate education on medication were 2.5 times more likely to rate the care as poor for PCC than those who felt adequately informed (AOR: 2.25; 95% CI: 1.67–3.90) (Table 5).

**Table 5:** Overall factors associated with person-centered care practices in public and private general hospitals.

		Poor	Good	COR (95%)	AOR (95%)
Type of the hospital	Private	123	300	**0.22 (0.164,0.29)**	**0.44 (0.26,0.75)**
	Governmental	277	148	1	1
Gender	Female	203	243	0.87 (0.66,1.14)	0.89 (0.60,1.32)
	Male	197	205	1	1
Marital status	Single	71	91	0.79 (0.51,1.23)	1.27 (0.65,2.47)
	Married	266	255	**0.59 (0.414,0.85)**	0.86 (0.51,1.45)
	Others	63	102	1	1
Educational level	No education	31	49	1.54 (0.89,2.67)	2.26 (1.01,5.04)
	Primary	136	143	1.02 (0.69,1.52)	1.57 (0.87,2.82)
	Secondary	157	178	1.10 (0.75,1.61)	1.37 (0.79,2.38)
	Higher	76	78	1	1
Insurance status	Insured	177	106	**2.56 (1.90,3.43)**	0.70 (0.41,1.18)
	Noninsured	223	342		1
Do you know each other with the physician who gave you the care	No	192	60	**5.97 (4.27,8.35)**	**4.80 (3.07,7.50)**
	Yes	208	388	1	1
Awareness of your disease or case	No	135	81	**2.31 (1.68,3.17)**	0.92 (0.52,1.64)
	Yes	265	367	1	1
Awareness of the treatment options	No	131	96	**1.79 (1.32,2.43)**	1.63 (0.94,2.82)
	Yes	269	352	1	1
Access services easily within the institution	Poor	251	103	**5.64 (4.18,7.61)**	**3.36 (2.24,5.03)**
	Good	149	345	**1**	1
Disturbing sound in the compound	Poor	116	92	**1.58 (1.15,2.17)**	1.06 (0.66,1.68)
	Good	284	356	1	1
The hospital's external attractive status	Poor	132	89	**1.99 (1.45,2.71)**	**1.65 (1.05,2.59)**
	Good	268	359	1	1
Privacy to access your care	Poor	277	58	**15.14 (10.69,21.45)**	**11.21 (7.38,17.04)**
	Good	123	390	1	1
The plan of care communicated to wish you enough	Poor	97	67	**1.82 (1.29,2.57)**	0.98 (0.59,1.62)
	Good	303	381	1	1
Information told on medication enough	Poor	171	84	**3.24 (2.38,4.41)**	**2.55(1.67,3.90)**
	Good	229	364	1	1

COR, crude odds ratio, AOR, adjusted odds ratio.

Bold: indicates statistical significance (*p* < 0.05).

## Discussions

This study assessed medical practices in Addis Ababa, Ethiopia, with a focus on aligning care with patient preferences and involving patients in decision-making. It included consultations with both patients and healthcare providers, ensuring that dignity and compassion were maintained throughout the treatment process, including in medication choices.

Overall, 52.8% of respondents rated healthcare practices (PCC) as good, aligning with findings from previous studies in Tigray and Addis Ababa, Ethiopia (6, 12). However, this finding is lower than those reported in previous studies conducted in Norway (86.5%), China (59.7%), and Saudi Arabia (73%) (7, 13–15). The disparity may be attributed to variations in socioeconomic levels, differences in study design and timing, and variations in healthcare systems. Socioeconomic factors can influence both patient expectations and access to care. Differences in study design and timing may affect how data is collected and interpreted. Additionally, variations in healthcare systems across regions can impact the implementation and effectiveness of care practices.

The subgroup analysis found that 70.9% of private hospital patients and 34.8% of public hospital patients rated person-centered care practices (PCC) as good. This aligns with a previous cross-sectional study conducted in Addis Ababa (6), which reported that 70.2% of private patients and 27.8% of public patients rated person-centered care practices (PCCP) positively. This difference likely stems from several factors. Private hospitals typically have better resources, higher staffing levels, and more personalized care due to greater funding, leading to higher patient satisfaction. In contrast, public hospitals face budget constraints, higher patient volumes, and limited resources, which can affect the quality of person-centered care (PCC) (16). Additionally, patients in private hospitals may have higher expectations, while those in public hospitals, dealing with more complex health issues, may rate their care differently.

Hospital ownership type (public or private) was identified as a significant factor influencing patient-centered healthcare practices, as supported by a previous study (7, 9, 17–20). This disparity may be due to private hospitals generally offering more advanced medical facilities and technology, shorter wait times, and better access to internal services compared to public hospitals (16). A statistically significant association was found between healthcare practices and hospital attractiveness, consistent with studies conducted in Addis Ababa (7). Patients likely feel more comfortable and have a better healthcare experience in hospitals with a welcoming atmosphere, which can lead to increased patient involvement and a better healthcare system.

The study found a statistically significant association between healthcare practices and privacy in accessing care, consistent with findings from a study conducted in Addis Ababa (6, 7). Patients may feel more comfortable sharing concerns when provided with privacy, such as private examination rooms or confidential communication, which enhances trust and open communication with healthcare professionals and supports care.

Information on the medication is a critical factor in healthcare practices, as supported by previous studies (6, 7, 21). Clear communication about medications enables patients to take an active role in their care. When informed about the purpose, correct usage, side effects, and interactions of their medications (22), patients can make better decisions, leading to improved adherence and outcomes. This approach respects patients' preferences, a core element of person-centered care, and fosters trust between patients and healthcare providers, promoting a collaborative treatment approach that prioritizes patient well-being and autonomy.

Personal and professional factors play a significant role. Research in Addis Ababa and central Ethiopia found that patients who felt familiar with their healthcare provider were 60% less likely to rate their interaction negatively (2, 3, 6). Additionally, a study in central Ethiopia showed that these patients were twice as likely to receive empathic care (7). The study also found a statistically significant association between perceived closeness to physicians and healthcare practices.

### The limitation of the study

This cross-sectional study assessed person-centered care practices, but factors such as participants' moods or personal issues during data collection may have influenced their responses, potentially affecting the accuracy of the findings. Additionally, the study was conducted in Addis Ababa, the capital city of Ethiopia, and its findings may not be easily generalized to other regions. Significant differences in infrastructure and the availability of skilled healthcare professionals across regions may limit the applicability of the results.

## Conclusion

Overall, 52.8% of respondents rated healthcare practices as good, with private hospitals in Addis Ababa (70.9%) showing a higher percentage of person-centered care practices compared to public hospitals (34.8%). Key factors influencing PCCs include hospital type, perceived intimacy with providers, ease of access, privacy, and medication information. We recommend targeted improvements in public hospitals to enhance the quality of PCC. Additionally, the findings should be considered in the context of hospitals in less urbanized areas of Ethiopia, where challenges in infrastructure and resources may affect PCC implementation.

## Data Availability

Publicly available datasets were analyzed in this study. This data can be found here: https://figshare.com/articles/dataset/The_data_for_Personcentered_care_practice_between_public_and_private_General_Hospitals_in_Addis_Ababa_Ethiopia/28001708?file=51086549.

## References

[1] Handley SC Bell S Nembhard IM. A Systematic Review of Surveys for Measuring Patient-centered Care in the Hospital Setting. Vol. 59, Medical Care. Philadelphia, PA: Lippincott Williams and Wilkins (2021). p. 228–37. 10.1097/MLR.0000000000001474 PMC7878319 33229897

[2] Tamiru B Beharu M Tesfaye T Belay Y. Scope of patient centered care practice in public hospitals of benishangul gumuze regional state. Qual Prim Care. (2017) 26(2017):31–7.

[3] Tamiru B. Facilitators and barriers of patient centered care practice in Public Hospitals of Benishangul Gumuze Regional State, South West Ethiopia. Rehabil Sci. (2021) 6(1):10. 10.11648/j.rs.20210601.12

[4] Kobrai-abkenar F Pourghane P Jafarzadeh-kenarsari F. Heliyon psychometric properties of the persian language person-centered climate questionnaire—patient version (PCQ-P) what does this paper contribute to the wider global clinical community? Heliyon. (2020) 6(October 2019):e05154. 10.1016/j.heliyon.2020.e05154 33088943 PMC7567042

[5] Ryan BL Brown JB Tremblay PF Stewart M. Measuring patients’ perceptions of health care encounters: examining the factor structure of the revised patient perception of patient-centeredness (PPPC-R) questionnaire. J Patient Cent Res Rev. (2019) 6(3):192–202. 10.17294/2330-0698.1696 31414031 PMC6675140

[6] Birhanu F YK Addis A Alemayehu D Shifera N. Patient-centered care and associated factors at public and private hospitals of Addis Ababa: patients’ perspective. Patient Relat Outcome Meas. (2021) 2:107–16. 10.2147/PROM.S301771 PMC8144361 34045910

[7] Rahel GE Biks GA Worku N Endalew B Dellie E. Patient-centered care and associated factors among adult admitted patients in South Wollo Public Hospitals, Northeast Ethiopia. Patient Prefer Adherence. (2022) 16:333–42. 10.2147/PPA.S346000 35173419 PMC8841686

[8] Kamimura A Weaver S Armenta B Gull B Ashby J. Patient centeredness: the perspectives of uninsured primary care patients in the United States. Int J Care Coord. (2019) 22(1):19–26. 10.1177/2053434519836424

[9] Kuipers SJ Cramm JM Nieboer AP. The importance of patient-centered care and co-creation of care for satisfaction with care and physical and social well-being of patients with multi-morbidity in the primary care setting. BMC Health Serv Res. (2019) 19(1):13. 10.1186/s12913-018-3818-y 30621688 PMC6323728

[10] Nigusie A Endehabtu BF Angaw DA Teklu A Mekonnen ZA Feletto M Status of compassionate, respectful, and caring health service delivery: scoping review. JMIR Hum Factors. (2022) 9(1):e30804. 10.2196/30804 35129450 PMC8863364

[11] Yoon JY Roberts T Grau B Edvardsson D. Person-centered climate questionnaire-patient in English: a psychometric evaluation study in long-term care settings. Arch Gerontol Geriatr. (2015) 61(1):81–7. 10.1016/j.archger.2015.03.010 25865746

[12] Berhe H BH Bayray A Godifay H Gigar G Beedemariam G. Status of caring, respectful and compassionate health care practice in tigrai regional state: patients’ perspective. Int J Caring Sci. (2017) 10(3):1118–28.

[13] Bergland Å Hofoss D Kirkevold M Vassbø T Edvardsson D. Person-centred ward climate as experienced by mentally lucid residents in long-term care facilities. J Clin Nurs. (2015) 24(3–4):406–14. 10.1111/jocn.12614 24787347

[14] Yang Y Li H Xiao LD Zhang W Xia M Feng H. Resident and staff perspectives of person-centered climate in nursing homes: a cross-sectional study. BMC Geriatr. (2019) 19(1):292. 10.1186/s12877-019-1313-x 31664918 PMC6819492

[15] Al-Sahli B Eldali A Aljuaid M Al-Surimi K. Person-centered care in a tertiary hospital through patient’s eyes: a cross-sectional study. Patient Prefer Adherence. (2021) 15:761–73. 10.2147/PPA.S286237 33883884 PMC8055245

[16] Luxford K Safran DG Delbanco T. Promoting patient-centered care: a qualitative study of facilitators and barriers in healthcare organizations with a reputation for improving the patient experience. Int J Qual Health Care. (2011) 23(5):510–5. 10.1093/intqhc/mzr024 21586433

[17] Ethiopian Ministry of Health. Health sector transformation plan II 2020/2021–2024/2025. Ethiop Minist Heal. (2021) 25:1–128.

[18] Arnetz JE Zhdanova L Arnetz BB. Patient involvement: a new source of stress in health care work? Health Commun. (2016) 31(12):1566–72. 10.1080/10410236.2015.1052872 27054396 PMC5028106

[19] Adesanya T Gbolahan O Ghannam O Miraldo M Patel B Verma R Exploring the responsiveness of public and private hospitals in Lagos, Nigeria. J Public Health Res. (2012) 1(1):2–6. 10.4081/jphr.2012.e2 25170439 PMC4140314

[20] Institute of Medicine (US) Committee on Quality of Health Care in America. Crossing the Quality Chasm: A New Health System for the 21st Century. Washington, DC: National Academies Press (US) (2001). 25057539

[21] Edvardsson D Watt E Pearce F. Patient experiences of caring and person-centredness are associated with perceived nursing care quality. J Adv Nurs. (2017) 73(1):217–27. 10.1111/jan.13105 27532230

[22] Singh S Evans N Williams M Sezginis N Baryeh NAK. Influences of socio-demographic factors and health utilization factors on patient-centered provider communication. Health Commun. (2018) 33(7):917–23. 10.1080/10410236.2017.1322481 28541816

